# The history of episodic memory

**DOI:** 10.1098/rstb.2023.0396

**Published:** 2024-09-16

**Authors:** Christoph Hoerl, Teresa McCormack

**Affiliations:** ^1^ Department of Philosophy, University of Warwick, Coventry, UK; ^2^ School of Psychology, Queen’s University Belfast, Belfast, UK

**Keywords:** episodic memory, time, self, history of psychology, mental time travel

## Abstract

Over the course of his research, Endel Tulving offered a number of somewhat different characterizations of episodic memory. Do they indicate that he changed his mind over time as to what episodic memory is, or did his core understanding of the nature of episodic memory stay the same? In this article, we offer some support for the latter claim, and in particular for thinking that, throughout his life, Tulving took as a defining feature of episodic memory the distinctive awareness of the self in time it involves. We argue that it is easier to see the continuities rather than the discontinuities in Tulving’s writings once their historical context is taken into account, where this involves both the authors who influenced his thinking, as well as the intellectual climate at the different times he was writing. We also discuss two recent bodies of work on episodic memory that take aspects of Tulving’s writings as their point of departure, but try to factor out into separate ingredients what he arguably saw as a unitary phenomenon. Considering aspects of the dialectic between them and Tulving’s view might shed further light on some of the motivations behind the latter.

This article is part of the theme issue ‘Elements of episodic memory: lessons from 40 years of research’.

## 1. Introduction

Endel Tulving’s monograph *Elements of episodic memory* opens with the following description of its subject matter:

Remembering past events is a universally familiar experience. It is also a uniquely human one. As far as we know, members of no other species possess quite the same ability to experience again now, in a different situation and perhaps in a different form, happenings from the past, and know that the experience refers to an event that occurred in another time and in another place. Other members of the animal kingdom can learn, benefit from experience, acquire the ability to adjust and adapt, to solve problems and make decisions, but they cannot travel back into the past in their own minds [1, p. 1].

Looking back at this description some 20 years later, Tulving [2, p. 6] writes: ‘Reading it [today], it is not difficult to see that it shows its age—I would not say exactly the same thing now. By and large, however, it does not seem to be terribly out of line’. Expanding on this comment, he mentions in particular the characterization of episodic memory as involving ‘mental time travel’. In the book, he claims, he used the phrase as a mere ‘device’ [2, p. 7] to help his readers see the difference between episodic recollection and other forms of memory. Subsequently, it was to become a concept much more central to his theorizing, embellished by further notions, especially those of ‘autonoetic consciousness’ and ‘chronesthesia’ [e.g 3–5].

Interestingly, *Elements of episodic memory* itself, in turn, harks back to Tulving’s very first characterization of episodic memory, yet another 10 years earlier, in the book chapter ‘Episodic and semantic memory’ [6]. Also here, too, we find Tulving expressing some sympathy, but also reservations, about his previous attempts at capturing what he meant by ‘episodic memory’. Indeed, a whole chapter of *Elements of episodic memory*, carrying the title ‘Inchoate distinction’, is devoted to the episodic/semantic distinction as first presented in 1972. In it, Tulving argues, for instance, that his original characterization may have underplayed similarities that also exist between episodic and semantic memory, or may too easily, and mistakenly, have been taken to imply that the distinction is exhaustive. Furthermore, he also discusses respects in which the adoption of the term ‘semantic memory’, to contrast with ‘episodic memory’, may have been less than happy.

One key exegetical question raised by comments such as these is thus whether they should be taken to indicate changes over time in how Tulving uses and understands the notion of episodic memory itself—perhaps there is more discontinuity than even his own remarks acknowledge—or whether his core understanding of that notion—of what episodic memory is—was the same throughout, even though it may have been articulated in different ways on different occasions.^1^

In this article, we aim to offer some considerations in support of a version of the latter hypothesis. In particular, we explore the idea that, from the start, Tulving conceives of episodic memory as having a characteristic dual experiential aspect, which finds its mature articulation (albeit perhaps not an ultimate clarification) in the two notions of ‘autonoesis’ and ‘chronesthesia’ that become prominent in his later descriptions of episodic memory. Part of our argument will be that it is perhaps easier to see the continuities rather than the discontinuities in Tulving’s thinking about episodic memory once the historical context is taken into account, where this involves both the authors who influenced his thinking, but also the intellectual climate at the different times he was writing.^2^

However, the scope of our article is not purely historical. Our emphasis on the continuities in Tulving’s writings stands somewhat in contrast to two recent bodies of work that can be seen to selectively appropriate only some elements of his work: one of them (sometimes associated with the idea of ‘what–where–when memory’) conceptualizes episodic memory as the retention of a certain kind of contextual information; the other as but one manifestation of a more general capacity for ‘mental simulation’ or ‘self-projection’. In each case, the claim is that factoring out (what is taken to be) a specific ingredient of Tulving’s evolving conception of episodic memory, but freeing it from others perceived to be idiosyncratic to him, yields a better understanding of the ‘core’ capacity at issue. Mapping out some aspects of the dialectic between the relevant approaches and what we take to be Tulving’s view might help, we hope, in bringing out what motivates the latter, and why some merit may remain in continuing to develop the kind of dual-aspect account of episodic memory it embodies.

## 2. The pre-history of episodic memory

As we will suggest, the dual concepts of *autonoetic consciousness* and *chronesthesia* can be seen as the mature articulations of two aspects of episodic memory that are central to Tulving’s thinking about episodic memory throughout. As he explains at one point, they are *aspects* of episodic memory especially in the sense that both notions are in fact intended to express ingredients of the same idea—that integral to episodic remembering is a *distinctive awareness of the self in time*:

[B]oth autonoesis and chronesthesia imply awareness of self in time, [although] the emphasis on self versus time is different in the two concepts: in autonoesis the emphasis is on awareness of self, albeit in subjective time, whereas in chronesthesia the emphasis is on awareness of subjective time, albeit in relation to self [4, p. 315].

While the terms ‘autonoetic consciousness’ and ‘chronesthesia’ emerge only later in Tulving’s writing, we believe that related ideas can already be seen to be present in his earliest writings on episodic memory. Consider, for instance, this key passage from his original 1972 chapter:

Episodic memory receives and stores information about temporally dated episodes or events, and temporal-spatial relations among these events. A perceptual event can be stored in the episodic system solely in terms of its perceptible properties or attributes, and it is always stored in terms of its autobiographical reference to the already existing content of the episodic memory store [6, p. 385].

While the first sentence of this passage is frequently cited in the literature on episodic memory,^3^ here we want to draw attention to the second, and in particular its own second half. What it suggests is that a defining feature of episodic memory for Tulving already at this stage is its distinctive relationship to the self in time, an idea he reinforces a few pages later by writing that ‘an integral part of the representation of a remembered experience in episodic memory is its reference to the rememberer’s knowledge of his personal identity’ [6, p. 389]. Yet, even though remarks along these lines are present in Tulving’s writings on episodic memory from the start, they initially do not have the prominence they increasingly assume roughly from the time of *Elements of episodic memory* onwards, when they also become much more unambiguously associated with experience. As we will see, Tulving himself—always an outspoken commentator of the state of the science of psychology—can be seen to give some reasons for this evolution in his writing about episodic memory.

### (a) Tulving, the philosophers, and Reiff and Scheerer on *remembrance* versus *memoria*

Remarks in both ‘Episodic and semantic memory’ and *Elements of spisodic memory* reveal an important underlying influence on Tulving’s research: the philosophical tradition of work on memory. In ‘Episodic and semantic memory’, Tulving writes that:

[t]he notion that memory takes different forms which must not be confused in analyses of phenomena of memory has a long history in the philosophical literature. Thus, the concepts of episodic and semantic memory are by no means new, although the traditional terminology tended to vary from writer to writer and although the philosophical categories of memory have had no influence on psychological research [6, p. 385].

The beginning of the main text of *Elements of episodic memory* returns to this theme, identifying precursors of the distinction between episodic and semantic memory in Bergson [15], Russell [16] and Furlong [17].^4^

Thus, in both 1972 and 1983, Tulving explicitly refers to the philosophical literature on memory as part of his own inspiration, a literature that he is clearly very familiar with. Two features of this literature, in particular, are noteworthy. The first is that the authors in question distinguish between different forms of memory on explicitly phenomenological grounds, i.e. on the basis of the distinctive types of conscious awareness they involve, just as Tulving will go on to do more and more prominently. The second is that it is not just the general distinction between episodic and semantic memory that is prefigured in the relevant philosophical writings in this context, but also Tulving’s specific emphasis on time and the self in connection with episodic memory. For Furlong, for instance, what he calls recollection, as contrasted with mere retentiveness, involves the twin features of one’s ‘mind look[ing] back to past events’ [17, p. 72] and ‘placing [onself] in [one’s] own position in the past context’ [17, p. 81], with the former happening by way of the latter.

We can perhaps get an even clearer picture of the tradition Tulving locates his own work in when we look at the one exception he mentions to his claim that ‘the philosophical categories of memory have had no influence on psychological research’ [6]. This is a chapter named ‘Forms of memory’ in the 1959 monograph *Memory and hypnotic age regression* by Robert Reiff and Martin Scheerer. Although the rest of the volume is mostly devoted to the somewhat specialized topic mentioned in its title, this chapter contains a largely self-contained general discussion of the nature of memory. In particular, Reiff and Scheerer discuss a distinction between two forms of memory that they call *remembrance* and *memoria*, respectively. As they explain:

[b]asically, memory is the retention of contents, events or activities in some form by the individual, either with or without the experience of a personal-temporal index. [F]or the purpose of clarification […] memories [can therefore be classified] into two primary forms: those *with* the experience of an autobiographic index, to be called *remembrances*; and those *without* the experience of an autobiographic index to be called *memoria* [20, p. 24f.].

Reading Reiff and Scheerer’s discussion against the background of Tulving’s 1972 chapter—which he later describes as ‘most directly influenced by Reiff and Scheerer’s ideas’ [1, p. 18]^5^—one thing that stands out is the central role they give to the notion of experience in framing their distinction. Perhaps most significantly, this also goes for their statement that ‘remembrances are always accompanied by the experience of personal continuity through time, while in memoria this experience is absent’ [20, p. 25], which Tulving quotes—with apparent approval—both in ‘Episodic and semantic memory’ [6, p. 389] and again in *Elements of episodic memory* [1, p. 18]. This is despite the fact (shortly to be discussed in more detail) that Tulving himself, in his early work, rarely uses the term ‘experience’ when speaking in his own voice.

In fact, Reiff and Scheerer’s work can be seen to anticipate a number of ideas that are not as yet fully spelled out in ‘Episodic and semantic memory’, but that will become increasingly prominent in Tulving’s subsequent writing. Thus, they point out that what they call memoria does not just comprise ‘items of acquired knowledge [such as] mathematical formulae, arithmetic, vocabulary, telephone numbers, etc’ [20, p. 26], but can also include knowledge, say, about the dates at which certain events happened. This is echoed by Tulving’s evolving understanding of semantic memory, which comes to include the idea that ‘[t]he semantic system handles temporal concepts as it does others’ [1, p. 42]. In both sets of authors, this reinforces the view that the crucial distinguishing feature of remembrance/episodic memory is not the information encoded as such but its specific experiential nature—the fact, as Tulving puts it, that ‘temporal relations of events […] are recorded experientially in subjective time’ [1, p. 42].

Playing an increasingly important role in Tulving’s work, and already prefigured in Reiff and Scheerer, is also the thought that empirical support for the idea of a distinct form of memory—marked by ‘personal temporality and inner representation of time’, as put by the latter [20, p. 29]—can be found in dissociations observed in amnesia. Reiff and Scheerer refer in this context particularly to work by Claparède [23], Katzaroff [24] and MacCurdy [25]. *Elements of episodic memory*, too, mentions Claparède on several occasions, but it was Tulving’s own work with patient N.N. [3] that would prove particularly significant for his work (see §3.2).

Tulving’s intellectual debt to Reiff and Scheerer is thus clear, and also explicitly acknowledged. This lends at least some support to the hypothesis that, from the start, the distinction Tulving wants to draw between episodic and semantic memory is in crucial part a phenomenological one, just as is the case with Reiff and Scheerer’s distinction between remembrance and memoria. His appeals to the philosophical tradition of writing about memory might be seen in a similar light. Conversely, if Tulving did intend his own episodic/semantic distinction to diverge in this important respect from that drawn by these claimed predecessors, it would be reasonable to expect to see some remarks to this effect in his work.

Nevertheless, in contrast, say, to Reiff and Scheerer, and also looking at the growing prominence of explicitly phenomenological themes in his own subsequent writings, Tulving is clearly initially reticent to employ the vocabulary of experience or awareness himself.^6^ If, as we are suggesting, it is in fact the same understanding of the episodic/semantic distinction that motivates Tulving throughout, why is he initially so reluctant to embrace it more explicitly and wholeheartedly? Another theme running through his writing might give some indications.

### (b) Tulving in his time

A striking feature of Tulving’s writings about episodic memory are his frequent side-swipes at the state of academic psychology, and in particular at what he perceives to be a long shadow cast by behaviourism. All the way back in ‘Episodic and semantic memory’, Tulving [6, p. 382] begins his discussion by speaking of ‘the limbo into which [the very term ‘memory’] was swept by the tide of behaviorism’. However, the main, more specific, theme that becomes particularly prominent in his later writings (e.g. [2,3]) is the legacy, lingering on even after the demise of behaviourism itself, of a hostility towards the topic of consciousness. As he writes in 1989, ‘[i]n this regard, cognitive psychology is rather similar to behaviourist and early functionalist approaches to psychology; all three represent ‘behaviouralism’, which can be contrasted with mentalism, the study of consciousness’ [26, p. 5].^7^

Given this conception of the intellectual climate that he was working in, it would therefore not be surprising to see Tulving’s own work on episodic memory, at least initially, exhibiting a certain ambivalence. For instance, his evidently positive view, already in the 1972 chapter, of Reiff and Scheerer’s work, has to be qualified by the fact that their approach was strongly influenced by the psychoanalytic tradition, whereas Tulving clearly saw himself writing for quite a different audience. This may explain some of the different framings he uses to introduce his distinction between episodic and semantic memory, even if it is intended to parallel their distinction between remembrance and memoria.

*Elements of episodic memory* [1], in particular, can be seen to be hovering between two different understandings of the proper domain of scientific psychology.^8^ Thus, despite its opening sentence (‘Remembering past events is a universally familiar experience’), experience hardly figures in the first part of the book. For instance, of the 25 pages making up the third chapter of the book, which aims to provide a systematic inventory of differences between episodic and semantic memory, a mere 13 lines are devoted to the topic of ‘Recollective experience’.

When he turns more explicitly to the topic of experience at the beginning of part II of the book, it is initially still not clear how exactly Tulving thinks of the role of science in its study. As he writes, ‘[t]he rememberer’s memory is phenomenal, the psychologist’s is objective’ [1, p. 123], and although he goes on to say that ‘an important task of the science of memory is to relate the two’ [1, p. 123], only a few pages later he worries that it ‘looks almost as if there was something basically incompatible between human cognition as seen by contemporary cognitive psychology and the human experience that characterizes one of the most advanced forms of cognition, episodic memory’ [1, p. 125].

As the book goes on, though, Tulving seems to become increasingly more assertive about the centrality of consciousness to the study of episodic memory, indeed concluding that the very reason ‘why cognitive psychology of memory has neglected subjective experience of remembering [is that] the question can meaningfully arise only in the study of episodic memory; it has not been raised, because cognitive psychology has not yet begun such study’ [1, p. 128f]. Some fifty pages later, the message could not be clearer: ‘In theories of episodic memory, recollective experience should be the ultimate object of interest, the central aspect of remembering that is to be explained and understood’ [1, p. 184].

What we are suggesting, then, is that something like this latter understanding of the nature of episodic memory is present in Tulving’s work from the start. What changes in his thinking over time is not this view of what episodic memory is, but his view of the potential capabilities of cognitive psychology in studying it. His initial formulations, in particular in his 1972 chapter, but also in some of the 1983 book, are shaped by the perceived need to bring the importance of the distinction between episodic and semantic memory home to an audience who, at that time, was largely hostile to talk about experience and consciousness. As his work develops, though, he increasingly comes to the view that any genuine attempt to recognize episodic memory for what it is has to start with its distinctive conscious phenomenology. To some extent, this coincided with a general shift within cognitive psychology towards an attitude more hospitable to the study of consciousness. In this respect, Andonovski [8, p. 42] seems right when he calls Tulving’s 1985 article ‘Memory and Consciousness’, in particular, ‘tone-shifting’. There, Tulving says:

After its banishment as an epiphenomenon by behaviouristic psychology, consciousness has recently again been declared to be the central problem of psychology. […] Nowhere is the benign neglect of consciousness more conspicuous than in the study of human memory. One can read article after article on memory, or consult book after book, without encountering the term ‘consciousness.’ Such a state of affairs must be regarded as rather curious. One might think that memory should have something to do with remembering, and remembering is a conscious experience [3, p. 1].

As an aside, given Tulving’s own acknowledgement of work in philosophy as an influence on his thinking, we may also note somewhat parallel developments philosophy underwent over the same period. When Tulving remarks, at the time of writing ‘Episodic and semantic memory’, that ‘the philosophical categories of memory have had no influence on psychological research’ [6, p. 385], it has to be said that some of the responsibility for this must also be sought in work in philosophy at the time. Apart from earlier examples from the history of the philosophy of memory, the one roughly contemporary work Tulving mentions is Stanley Munsat’s [28] *The concept of memory*. This is a work steeped in the tradition of ‘ordinary language philosophy’—according to which philosophical questions can be resolved (and often dissolved) by careful attention to the way (English-speaking) people talk.^9^ As such, it represents a philosophical approach that is very much at odds with the idea of a potentially productive dialogue between philosophy and psychology. Indeed, the philosophy of memory, too, by and large did not rediscover questions about the phenomenology of memory until closer to the end of the twentieth century.

## 3. Tulving’s legacy

We have looked at the role in Tulving’s writings on episodic memory played by the idea that episodic memory involves a distinctive form of awareness of the self in time. While perhaps not initially articulated in just those terms, we have tried to offer some support for the suggestion that an idea along those lines is present in Tulving’s work from the start and, with time, becomes increasingly more explicit.

In what follows, we will switch from considering Tulving’s own writings on episodic memory to two recent developments in the literature on episodic memory that take as their avowed point of departure (what are claimed to be) elements of Tulving’s work. One strand of this recent work is associated with a conceptualization of episodic memory as ‘what, where, when’ memory; the other conceives of episodic memory as just one species of a more general phenomenon of ‘mental simulation’. Very schematically, each of these two approaches might be seen as appropriating elements of one of Tulving’s dual notions of chronesthesia and autonoetic consciousness, respectively, while downplaying the significance of the other—thus departing from Tulving’s view that the two notions capture two aspects of episodic memory that are inextricably linked and cannot be understood in isolation from each other.

We can only provide a brief discussion of these two recent approaches to episodic memory in what follows—they are discussed more comprehensively elsewhere, and anyway our discussion of them does not aspire to any originality.^10^ They are relevant to the present paper primarily in so far as some of the issues they raise arguably trace back to the theme common to them, which is the idea that what Tulving sees as a unitary phenomenon—as encapsulated in the notion of an awareness of the self in time (see the beginning of §2, above)—can be factored out into separable components.^11^ As such, consideration of these issues might also help, in turn, to bring out some of the thoughts that animate Tulving’s original vision.

### (a) The idea of ‘what, where, when’ memory

As mentioned above, the phrase most frequently quoted from Tulving’s 1972 chapter is that episodic memory stores ‘information about temporally dated episodes or events, and temporal-spatial relations among these events’ [6, p. 385]. Indeed, Tulving’s phrase is often seen as an initial attempt at a *definition* of episodic memory, according to which episodic memory amounts to what has since also been referred to—particularly in the literature in comparative psychology—as ‘what, where, when’ memory [10]. A representative statement of this view can be found in in Pauline *et al*. [36, p. 3], who speak of a ‘content behavorial criterion of episodic-like memory’, which is

based on Tulving’s original definition (i.e., ‘a system that receives and stores information about temporally dated episodes or events, and temporal-spatial relations among these events’ [6]). This definition means that an episodic memory provides information about ‘what’ happened as well as ‘when’ and ‘where’ it happened. [36, p. 3]

It may be useful to distinguish between exegetical and substantive issues at this point. In line with the authors’ claim that the phrase they quote from Tulving constitutes his ‘original definition’ of episodic memory, it has become common to regard Tulving to have initially held a view according to which what makes memories episodic is that they encode a particular kind of information—specifically, contextual information about the time and place of occurrence of the remembered event.^12^ The material we discussed in the first half of the chapter makes this interpretation, at least if left unqualified, difficult to uphold. As we noted, for instance, the phrase from Tulving quoted by Pauline *et al*. is followed immediately by a sentence according to which an ‘autobiographical reference’, too, is essential to episodic memory. We have also discussed other aspects of Tulving’s 1972 chapter which suggest that he took what he later called ‘an awareness of self in time’ to be a defining feature of episodic memory from the start.

Setting this aside, though, it might nevertheless be thought that the quoted phrase—‘a system that receives and stores information about temporally dated episodes or events, and temporal-spatial relations among these events’ [36, p. 3]—describes something like the ‘core’ of episodic memory, to which Tulving added further, phenomenological, elements that may, e.g. only apply to or be demonstrable in human beings, and that are in principle separable from the supposed ‘core’. In other words, the thought would be that the phenomenon Tulving identifies in human beings can, in principle, be factored out into a ‘what, where, when’ element and some further additional elements that are to do with a distinctive conscious awareness of the self and time.

To use what is sometimes regarded as a litmus test for proposed definitions, the key challenge for the idea that the core of episodic memory is constituted by ‘what–where–when’ memory is that this description seems to provide neither necessary nor sufficient conditions for something’s being an episodic memory.^13^ We have already come across a version of the point about sufficiency in some of Reiff and Scheerer’s comments on what they describe as the distinction between remembrance and memoria. As they explain, memoria (which can be taken to be a precursor notion to semantic memory) can and often does include the retention of knowledge about particular, dated, events in the past. Conversely, to move on to the point about necessity, the ability to date an event can also not serve as a criterion for distinguishing episodic from semantic memory because human episodic memories are often not accompanied by a clear idea of ‘when’ the relevant event happened.

To be sure, the recollection of an event may often prompt an individual to ask themselves questions about when the event in question happened and to attempt to determine this by a variety of different means [38]. This might be seen to reinforce Tulving’s insistence, throughout his writings, on an intimate connection between episodic memory and the awareness of time (though see below for a somewhat different perspective). However, it also shows that the latter awareness cannot be reduced to being a matter of possessing information about something like a date at which the remembered event happened. As Tulving puts it in *Elements of episodic memory*, ‘each event in the episodic system is referred to a particular instant, date, or period in time. But the referent is not chronological time or calendar time; rather, at the occurrence of the event, it is the rememberer’s personally experienced time, and at recollection, his personal past’ [1, p. 39].

### (b) Episodic memory as simulation

A second strand of recent work on memory we want to discuss briefly takes as its point of departure especially some aspects of Tulving’s notion of ‘mental time travel’. Only mentioned in passing at the beginning of *Elements of episodic memory*, this notion assumes more central theoretical significance in Tulving’s 1985 article ‘Memory and consciousness’ [3] and subsequent work. Instrumental in this (as well as in the development of the related notion of autonoetic consciousness) were conversations—some reproduced in that article—which Tulving had with a densely amnesic patient identified by him as N.N. (elsewhere referred to under his real initials, K.C.). What these conversations showed was that N.N., apart from lacking any episodic memories for events he had experienced in the past, was also unable to envisage any events he might experience in the future. This led Tulving to hypothesize that episodic memory was in fact the manifestation of a broader capacity. A healthy person, Tulving says [3, p. 5], ‘is capable of mental time travel, roaming at will over what has happened as readily as over what might happen, independently of physical laws that govern the universe’; N.N., by contrast, lacked this capacity.

The idea of the existence of such a general capacity for ‘mental time travel’ subsequently became a major theme for research, which for instance demonstrated that common neural structures were active in both remembering the past and anticipating the future [39,40]. In the context of this research, however, some authors started to broaden the conception of the relevant capacity even further to also include, e.g. certain purely imaginative exercises amongst its manifestations [41,42].

Donna Addis [43, p. 233] provides a particularly stark expression of this way in which some theorists have moved on from Tulving’s understanding of a cognitive system facilitating ‘mental time travel’ when she writes that:

‘time’ and ‘travel’ may not be [its] defining, or even essential, features. Rather, it is the ‘mental’ rendering of experience that is the most fundamental function of this simulation system enabling humans to re-experience the past, pre-experience the future, and also comprehend the complexities of the present.

In other words, the fundamental cognitive capacity, for Addis, is an entirely generic capacity for generating ‘simulations’, or what has also been referred to a capacity for ‘self-projection’ [44]—mentally projecting oneself into different situations. Conversely, on this kind of view, episodic recollection—constituting one specific exercise of that generic capacity—can be factored out into two components: a ‘content’ delivered by the simulation system—which in and of itself is neutral, e.g. as to whether it represents an event that took place in the past,^14^ and which can therefore also be present outside episodic memory—plus some further, separate, ingredient that can ground the more specific judgement that the event thus represented is in fact a personally experienced past event.^15^

We will focus in particular on the purported second ingredient, which supposedly distinguishes between episodic recollection and other cognitive activities on this view, and which has been discussed under a number of different names, including ‘feeling of pastness’. One specific thought that has been gaining traction is that work on ‘metacognitive’ or ‘epistemic’ feelings might provide the materials for fleshing out this notion. What has attracted particular attention in this context are experiments in which memory judgements can be influenced by manipulating the fluency of cognitive processing, leading to the thought that such fluency might be one potential source of the ‘feeling of pastness’ (see [48] for a statement of a view along those lines). Very crudely speaking, the proposal is that, in episodic memory, a simulation of an event comes to be regarded as a representation of an event that was experienced in the past in virtue of a characteristic feeling accompanying the simulation, which in turn corresponds features of processing such as the fluency with which the simulation is produced.

In evaluating this idea, it is interesting to note that there has also been recent work on epistemic or metacognitive feelings in connection with memory that has put them to a somewhat different explanatory purpose. Dokic [49, p. 3], for instance, while speaking of an ‘episodic feeling’, clarifies that he means by this a feeling that accompanies an ‘experience [which] is episodic in virtue of facts independent of the episodic feeling’. He thinks of the role of that feeling in terms of the question as to what allows a person to determine, of a mental state that purports to be about a past event, whether it is a memory or not. Teroni [50] puts forward a feeling-based account of what he calls ‘memory identification’ that is most naturally read in a similar way. Indeed, on his account, it is the same feelings that underlie memory identification in the case of episodic as well as semantic memory. Both of these proposals are in line with a general account of epistemic feelings that sees their role as one of tracking the presence and performance of a given cognitive capacity, rather than as being part of what constitutes the relevant capacity in the first place [51].

The question is thus whether, in the case of episodic memory, feelings might also, in addition, be able to play the more specific explanatory role sketched above, of accounting for the way in which episodic recollection itself, distinctively, involves locating the remembered event in the past,^16^ thus making it different from other purported forms of cognition involving ‘simulation’. We cannot settle this question here, but the answer is likely to turn crucially on the extent to which one goes along with Tulving’s view that episodic memory itself has to be seen as an expression of the ‘capability […] that makes thinking about subjective time possible’ [4, p. 313], which he calls chronesthestia. To the extent that there is such a thing as a ‘feeling of pastness’—even on accounts that endorse the notion—it arguably already presupposes the existence of such a capability, because it is the result of an attribution.^17^ To use a phrase employed by David Pears [52, p. 238], such a feeling can suggest something about the past only to those who already know about the past ‘at closer quarters’. On a conception such as Tulving’s, by contrast, it is precisely such knowledge of the past at close quarters that episodic memory is seen as providing us with in the first place.

## 4. Returning to Tulving

Admittedly, we can only provide the barest sketch of how we conceive of some of the dialectic between Tulving’s own view of episodic memory and the two approaches discussed in the preceding section. Crudely speaking, one of them can be seen to be selectively appropriating ideas more often associated with Tulving’s earlier writings, the other with his later ones. Somewhat in contrast with this outlook, we have, in §2, emphasized continuities, rather than discontinuities, in Tulving’s thinking about episodic memory over time. Plausibly along with this goes the idea of a vision of episodic memory, present throughout his writing, as a unitary phenomenon, which cannot be factored out into different ingredients in the manner envisaged, in two different ways, by each of the two approaches we discussed in §3. Obviously, Tulving’s repeated expressions of frustration with his own attempts at characterizing episodic memory, as mentioned in §1, and the difficulties of interpretation researchers have raised with some of the terminology he settles on—in particular the dual notions of autonoetic consciousness and chronesthesia—demonstrate the challenge in spelling out what exactly such an alternative, unitary conception of episodic memory might come to. However, we hope to have given at least some reasons as to why this challenge we have inherited from Tulving might continue to be worth pursuing.

## Data Availability

This article has no additional data.

## Footnotes

^1^
Tulving clearly changed his mind in some respects. In 1972, he writes that ‘[n]othing very much is lost at this stage of our deliberations if the reality of the separation between episodic and semantic memory lies solely in the experimenter’s and the theorist’s, and not the subject’s mind’ [6, p. 384]. In later writings, he defends a much more robust view of episodic memory as ‘subserved by a distinct neurocognitive system that has evolved specifically for that purpose’ [5, p. 332].

^2^
For two excellent existing discussions of the historical development of Tulving’s thought and its legacy see Renoult & Rugg [7] and Andonovski [8].

^3^
Indeed, it is frequently read as having been intended by Tulving as a *definition* of episodic memory (see also below). For claims broadly along those lines, see Boyle [9], Clayton & Dickinson [10], Crystal [11], Hsieh & Ranganath [12], Michaelian *et al*. [13], Russell & Hanna [14].

^4^
Herrmann [18] identifies no fewer than 14 precursors to Tulving’s distinctions in the philosophical literature, allegedly going back as far as Aristotle [19].

^5^
Tulving also mentions in passing two other influences: a textbook chapter by T. A. Ryan [21], which he says he had to read as a teaching fellow of E. G. Boring, one of the book’s editors, and a chapter by Jerome Bruner, which features a distinction between ‘what may be called *memory with record*, where specific events are recoverable, and *memory without record*’ [22, p. 254].

^6^
Some alternative phrases he comes up with instead—speaking, e.g. of a ‘reference [integral to episodic memory] to the rememberer’s knowledge of his personal identity‘ [6, p. 389]—seem hardly more perspicuous, though.

^7^
Here and elsewhere, Tulving references Leahey’s [27] historical analysis of the development of psychology in the twentieth century.

^8^
In 2002, referring to the book, Tulving remarks that ‘[b]ack in 1983, […] the study of human memory was still trying to shake the powerful behaviourist or behaviouralist […] influences of the past’ [4, p 1]. He may plausibly have intended this remark to apply, at least in part, to himself too.

^9^
Munsat’s title is intended as an explicit nod to Gilbert Ryle’s [29] *The concept of mind*, one of the classic exemplars of the genre, and one of his main interlocutors is Norman Malcolm [30], another of its key proponents.

^10^
For some relevant discussions, see Boyle [31], Aranyosi [32,33], Phillips [34], Hoerl [35].

^11^
As will hopefully become evident, since Tulving sees the two types of awareness as interdependent with each other, the conception of the individual components that, according to each of the two approaches, make up episodic memory, consequently also differ significantly from him.

^12^
See footnote 3, above, for references to a number of papers where a claim along those lines is made.

^13^
Admittedly, Tulving himself never provides a definition cast in terms of necessary and sufficient conditions either. We are using this framing simply because it serves to make particularly vivid that, if there is a connection between episodic memory and time, it does not seem to consist in the former representing a particular type of *temporal information*. For more detailed discussion, see Boyle [31], Phillips [34], Suddendorf & Busby [37].

^14^
Cf. also, e.g. De Brigard & Gessell [45], whose paper is aptly titled ‘Time is not of the essence’, or Mahr *et al.* [46, p. 12], who claim that ‘episodic contents are not intrinsically temporal in nature. That is, episodic simulation does not seem to generate ‘past episodes’ and ’future episodes’’.

^15^
Dokic [47], accordingly, speaks of ‘two-tiered’ accounts of episodic memory. Hoerl [35] uses the label ‘two-factor accounts’.

^16^
Amongst other things. On feeling-based accounts such as that put forward by Perrin *et al*. [48], there are in fact a number of ‘components’ of the relevant feeling in addition to the one we focus on here (see [48, p. 3]).

^17^
See Perrin *et al*. [48, p. 9] or Boyle’s [31, p. 2520] claim that ‘there is nothing intrinsically ’past-ish‘’, about the purported feeling of pastness.
